# Adipose Decellularized Matrix: A Promising Skeletal Muscle Tissue Engineering Material for Volume Muscle Loss

**DOI:** 10.34133/bmr.0174

**Published:** 2025-04-17

**Authors:** Zimo Wang, Wei Liang, Rigele Ao, Yang An

**Affiliations:** Department of Plastic Surgery, Peking University Third Hospital, Beijing 100191, China.

## Abstract

Volume muscle loss is a severe injury often caused by trauma, fracture, tumor resection, or degenerative disease, leading to long-term dysfunction or disability. The current gold-standard treatment is autologous muscle tissue transplantation, with limitations due to donor site restrictions, complications, and low regeneration efficiency. Tissue engineering shows potential to overcome these challenges and achieve optimal muscle regeneration, vascularization, nerve repair, and immunomodulation. In the field of muscle tissue engineering, skeletal muscle decellularized matrices are regarded as an ideal material due to their similarity to the defect site environment, yet they suffer from difficulties in preparation, severe fibrosis, and inconsistent experimental findings. Adipose decellularized matrices (AdECMs) have demonstrated consistent efficacy in promoting muscle regeneration, and their ease of preparation and abundant availability make them even more attractive. The full potential of AdECMs for muscle regeneration remains to be explored. The aim of this review is to summarize the relevant studies on using AdECMs to promote muscle regeneration, to summarize the preparation methods of various applied forms, and to analyze their advantages and shortcomings, as well as to further explore their mechanisms and to propose possible improvements, so as to provide new ideas for the clinical solution of the problem of volume muscle loss.

## Introduction

Volume muscle loss (VML) is an irrecoverable injury with a muscle loss of more than 20%, as a result of trauma, fracture, tumor resection, or degenerative disease, often resulting in long-term dysfunction or even disability [1]. A large number of patients experience muscle loss due to fractures each year and suffer heavy financial burdens as a result [2]. Surgical reconstruction is often required due to extensive skeletal muscle loss that exceeds the limited autoregenerative capacity, and autologous muscle tissue transplantation is the current gold standard [1]. However, the results of autologous muscle tissue transplantation are not optimal as a result of the limited volume of muscle in the donor site, the prevalence of complications such as infections and necrosis, the inefficiency of regeneration at the recipient site, and the difficulty in restoring skeletal muscle function [3]. Successful treatment requires substantial muscle regeneration to replace lost tissue, adequate vascularization to supply blood flow, and nerve regeneration to allow repair of function, as well as favorable immunomodulation to reduce fibrosis [4]. The enormous demand, the inadequacy of existing treatments, and the stringent criteria for successful treatment have rendered the repair of large muscle defects a clinical challenge and an active field of research [5]. The search for an ideal restoration is therefore extremely urgent for VML.

Tissue engineering has been applied to a wide range of tissues and organs by combining seed cells, scaffolding materials, and bioactive factors to reconstruct tissues and organs in a laboratory setting [6], and this approach can effectively avoid the shortcomings of the current VML treatments and thus has great potential for application in this field [4]. Optimal tissue-engineered materials are crucial for regenerative effect, and decellularized matrices (dECMs) are increasingly favored by researchers, as they can mimic the microenvironment of the original tissue growth due to the preservation of the extracellular matrix (ECM) and the growth factors necessary for regeneration [7]. Moreover, the lack of cellular components reduces the immunogenicity of the material, which can effectively avoid the poor repair effect caused by the immune reactions [8]. dECMs from different sources have been used in preclinical and clinical trials and have been shown to have the potential to restore skeletal muscle function, and they include small intestinal submucosal dECMs, bladder dECMs, skeletal muscle dECMs (SMdECMs), and adipose dECMs (AdECMs) [9].

**Fig. 1.**
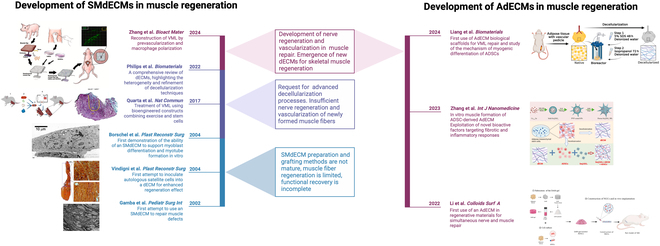
The development of skeletal muscle decellularized matrices (SMdECMs) and adipose decellularized matrices (AdECMs) in muscle regeneration. This figure shows a timeline of the development of SMdECMs and AdECMs in muscle regeneration, lists the most representative experiments at each stage, and summarizes the problems faced at each stage [16,18–20,22,107–110]. Reproduced with permission [16]: © 2002, Springer Nature. Reproduced with permission [18]: © 2022 Published by Dove Medical Press. Reproduced with permission [19]: © 2022 Published by Elsevier B.V. Reproduced with permission [20]: © 2024 Elsevier Ltd. Reproduced with permission [107]: © 2023 The Authors. Reproduced with permission [108]: © 2017, The Authors. Reproduced with permission [109]: © 2004 American Society of Plastic Surgeons. Reproduced with permission [110]: © 2004 American Society of Plastic Surgeons. VML, volume muscle loss; HUVECs, human umbilical vein endothelial cells; ADSCs, adipose-derived stem cells; SDS, sodium dodecyl sulfate; DAM-gel, decellularized matrix hydrogel; ECs, endothelial cells; hMuSC, human muscle satellite cells.

An SMdECM is ideal for the regenerative repair of large muscle defects due to having the same cellular microenvironment as the defective site, and there have been numerous experiments confirming the usefulness of its muscle regeneration [10–12], with a few exceptions [13–16]. In recent years, scholars have increasingly directed their attention toward adipose tissue and AdECMs with the objective of broadening the range of materials available for muscle regeneration (Fig. 1). It has been demonstrated that autologous adipose tissue reduces fibrosis and inflammatory response in the reconstruction of VML [13,17]. Zhang et al. [18] demonstrated that AdECMs combined with adipose-derived stem cells (ADSCs) have the potential to promote myogenic differentiation of ADSCs and the combination of Se@SiO_2_ can reduce oxidative stress. Li et al. [19] prepared injectable adipose decellularized hydrogel scaffolds loaded with ADSCs for the regeneration of peripheral nerves as well as structural and functional restoration of innervated skeletal muscles. Liang et al. [20] verified that perfusable adipose decellularized ECM bioscaffolds with ADSCs and L6 co-recellularization promote functional skeletal muscle regeneration.

The above experiments confirmed that AdECMs have the potential to serve as an alternative material choice for muscle regeneration; in addition, AdECMs have the following advantages over SMdECMs for VML: (a) The relatively loose structure of large adipose tissues compared to that of an SMdECM makes both the immersion and perfusion decellularization methods simple and reduces damage to the natural structure. (b) An AdECM is more potentially applicable for its abundant sources and sufficient yields and is easier to clinically transform. (c) Adipose tissue has monovascular blood supply, which makes it easier to anastomose with blood vessels in the body after transplantation and ensures the metabolic needs of cells in the scaffolds [20]. (d) An AdECM has the potential to reduce fibrosis and inflammatory responses and achieve better experimental consistency and reproducibility due to its composition and intrinsic molecular mechanisms with surrounding tissues [21].

An AdECM is an ideal material for tissue engineering, yet its potential for application in muscle tissue regeneration is still not fully discovered. The aim of this review is to summarize the relevant studies on using AdECMs to promote muscle regeneration, to summarize the preparation methods of various applied forms, and to analyze their advantages and shortcomings, as well as to further explore their mechanisms and to propose possible improvements, so as to provide new ideas for the clinical solution of the problem of VML.

## Regeneration Process of Skeletal Muscle

The process of skeletal muscle regeneration is explained in detail in a review by Philips et al. Briefly, the process of skeletal muscle regeneration consists of 5 highly orchestrated stages, including degeneration, regeneration, remodeling, and maturation (Fig. S1) [22]. The prominent feature of degeneration phase is the necrosis of muscle fibers and the recruitment of macrophages and muscle satellite cells (SCs) [23–26]. Collagen types I and III provide mechanical support and cell adhesion sites, while fibronectin and laminin enhance cell adhesion and migration, supporting SC differentiation [27,28]. Proteoglycans bind growth factors, regulating cell proliferation and differentiation [29]. In the regeneration site, SCs are activated and differentiate into myoblasts, which is accompanied by macrophage polarization from the pro-inflammatory subtype M1 to the anti-inflammatory subtype M2 [8,23,24,30–32]. The remodeling phase is associated with the contractile properties of myoblasts and vascularization [33–36]. ECM components like collagen VI aid in SC self-renewal and ECM remodeling [37]. Growth factors such as fibroblast growth factor, vascular endothelial growth factor (VEGF), and insulin-like growth factor (IGF) retained in a dECM promote cell proliferation and survival, facilitating vascular sprouting and functional recovery [22]. During maturation, reinnervation and neuromuscular junction formation are supported by the structural and biochemical cues provided by the dECM, creating a favorable environment for muscle regeneration [22,38].

## Component Comparison of SMdECMs and AdECMs

Previous studies have reported both overlap and differences in the composition of SMdECMs and AdECMs [39–42], which may suggest a potential mechanism by which both have myogenic effects but different effects. It should be clear that the components retained in the dECMs of the same tissue differ under different decellularization methods, and therefore, the myogenic effects of SMdECMs and AdECMs should correspond to the specific decellularization method. The present review summarizes only the components of the 2 sources of dECMs that have been reported by previous studies to elucidate the potential myogenic effects of AdECMs (Table S1).

## Methods of Preparing AdECM-Derived Scaffolds for Skeletal Muscle Regeneration

### Sources of AdECMs

**Fig. 2.**
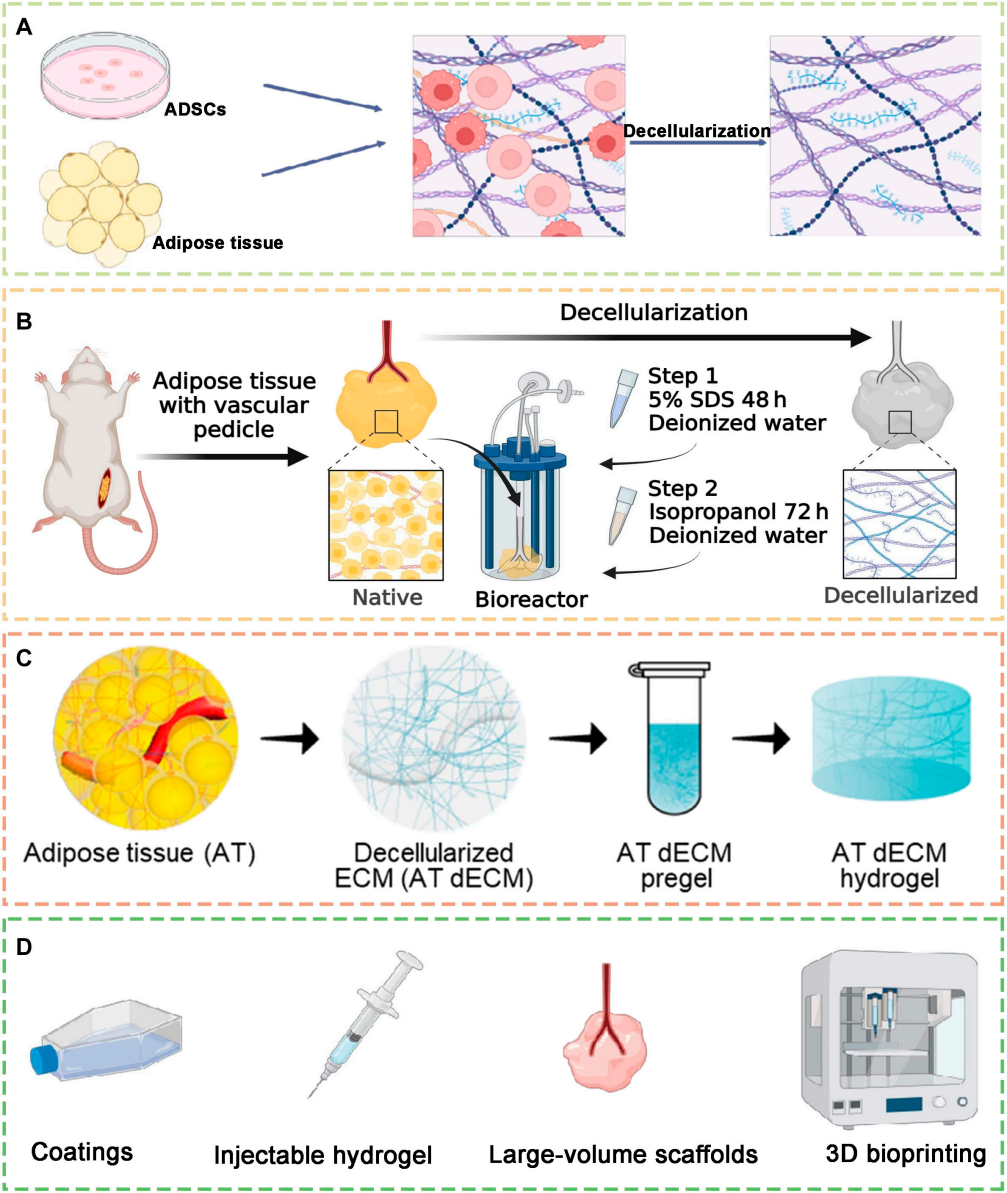
Sources and application forms of AdECMs. (A) An extracellular matrix was obtained by culturing ADSCs or harvesting adipose tissue and subsequently decellularizing them to obtain an AdECM. (B) Preparation and application of decellularized large-volume scaffolds [20]. (C) Key steps of DAM-gel (decellularized adipose extracellular matrix) preparation [63]. (D) Four forms of application of AdECMs reported in previous studies. Reproduced with permission [20]: © 2024 Elsevier Ltd. Reproduced with permission [63]: © 2023 Acta Materialia Inc. 3D, 3-dimensional.

AdECMs include both cell-derived and tissue-derived AdECMs (Table S2). Cell-derived AdECMs refer to dECMs deposited by ADSCs, which are cultured in extraction buffer followed by the addition of phosphate-buffered solution (PBS) to wash away the residual buffer and ultimately applied as a hydrogel. Tissue-derived AdECM preparation starts with obtaining adipose tissue from the human or animal body, followed by decellularization using physical, chemical, or enzymatic methods and subsequent preparation in the form of hydrogels, coatings, large-volume scaffolds, bioinks, etc., depending on the application scenario (Fig. 2).

The limited amount of AdECMs deposited by ADSC secretion and the protracted preparation time complicate the fulfillment of the therapeutic demand of VML. Conversely, AdECMs from adipose tissue sources are abundant and more readily available and therefore have a higher potential for application in both animal and clinical trials.

### Decellularization methods of AdECMs

Preparation of an AdECM comprises a variety of methods, including physical, chemical, and biological enzymes, and researchers often use different methods in combination to improve decellularization efficiency (Fig. S2A). Decellularization usually starts with physical methods. The destruction of the cellular structure is achieved by putting adipose tissue through imitative freeze–thaw cycles, high-speed agitation, and mechanical shredding or grinding. Chemical methods use hypotonic or hypertonic solutions to cause cell lysis through osmotic pressure and further use ionic and deionized detergents to remove lipids and cellular debris from the ECM, in addition to the application of organic solvents such as isopropanol, ethanol, or acids and bases for extraction to further dissolve cytoplasmic components and remove excess lipids, and the commonly used biological enzymes, including lipases, protein hydrolases, and nucleases, are able to achieve lysis of cellular components and removal of residual lipids, but when used alone, they are often inefficient in partitioned cells [39,42–44].

The methods of skeletal muscle decellularization have been summarized in detail in previous literature [22]. One of the most commonly used skeletal muscle treatments was established at the University of Padua. Incubation in 4% sodium deoxycholate (SDC) for 4 h was followed by DNase I for 3 h [11,14,16,45–49]. However, there is no uniform conclusion as to whether this method can be applied to human muscle tissue. Sodium dodecyl sulfate (SDS) shows higher decellularization efficiency; however, it has a higher risk of damaging the ECM microstructure [50]. When SDS concentrations were as low as 0.1% in combination with 0.1% trypsin and DNase/RNase treatment, there was still a large amount of collagen and glycosaminoglycan disruption [51]. In contrast, Triton X-100 is a gentler detergent than SDS, causing less damage to collagen, and combining it with trypsin improves penetration and thus decellularization efficiency [52]; however, the optimal concentration for decellularization of human muscle tissue remains inconclusive [53]. Researchers are constantly improving previous methods to optimize the efficiency of decellularization of skeletal muscle while preserving the maximum ECM composition and structure; however, the search for optimal solutions still requires extensive experiments on human skeletal muscle (Table S3).

The natural structure of skeletal muscle also dictates more demanding decellularization conditions compared to those for adipose tissue, as confirmed by the scarcity of decellularization experiments on human skeletal muscle [54]. Increased penetration of detergents means higher concentrations, with attendant damage to important ECM components such as collagen and glycosaminoglycans. Optimization of traditional methods by researchers poses other problems such as high costs and complex experimental steps. Therefore, until the ideal skeletal muscle decellularization method is established, AdECMs are important for myogenic muscle experiments.

### Recellularization methods of AdECMs

Comparing the previously reported methods of recellularization of SMdECMs and AdECMs, the recellularization of SMdECMs is more varied, including muscle stem cells, adipose stem cells, bone marrow mesenchymal stem cells, and vascular endothelial cells. In addition, skeletal muscle recellularization methods are more abundant and are mainly divided into 2 categories: in vitro and in vivo (Fig. S2B and C). The in vitro methods include seeding, injection, and perfusion using bioreactors, while the only currently reported in vivo method is injection.

The following analysis addresses the advantages and disadvantages of recellularization methods that have been applied in experiments of AdECM muscle regeneration. In vitro culture is simple to perform and can directly inoculate cells onto the surface of the cell matrix, which is suitable for rapid attachment and proliferation. However, this method is prone to the problems of uneven distribution of cells and low attachment efficiency, leading to the loss of cells, and it can be used only for in vitro experiments [55].

The hydrogel encapsulation method provides better protection against mechanical damage. In addition, the fluidic nature of a hydrogel allows for even distribution within the matrix, thereby improving the uniformity of cell distribution [56,57]. However, this method demands high performance of the hydrogel. It is imperative to optimize the biocompatibility of the hydrogel to avoid deleterious effects on cells [57]. Furthermore, it is imperative that the mechanical properties of hydrogels mimic those of natural tissues, as discrepancies in these properties can adversely impact cell function [58].

Injection into the scaffold pedicle offers several advantages, including the capacity to leverage the native vascular network of the tissue to achieve uniform distribution of cells and further promote the integration of cells with host tissues [20]. This method is particularly well suited for larger volumes of tissues or organs, especially in the context of VML. However, it is important to note that this approach may potentially compromise the vascular network during the perfusion process and necessitates anastomosis with blood vessels, thereby requiring a high level of surgical expertise. Furthermore, inadequate regulation of perfusion pressure and duration can potentially compromise cell viability [20].

The direct in vivo injection method has been utilized in SMdECM muscle regeneration. Similar to in vitro culture, the direct in vivo injection method is characterized by its simplicity and direct injection of cells into the target site, leveraging the in vivo environment to achieve cell distribution and integration. However, the distribution of cells may be nonuniform, particularly in larger tissues, which can potentially impact cell survival and the efficacy of the repair process [59].

Conversely, the bioreactor infusion method employed for SMdECMs holds promise for AdECMs, provided that it can overcome the challenges posed by the high equipment and technological demands, complexity of operation, and increased cost. The bioreactor infusion method has the potential to enhance cell permeation and homogeneous distribution, particularly in complex or larger tissues, by regulating parameters such as fluid dynamics, perfusion rate, and pressure [60]. Furthermore, bioreactors can better simulate the in vivo environment, improve cell survival, and better mimic the physiological state of cells in natural tissues through dynamic culture, thus promoting cell growth and functional expression [61,62].

In summary, for muscle regeneration of VML, direct injection into the scaffold pedicle is a superior option, and injection into a bioreactor is a commendable approach for AdECMs, as both allow for uniform cell penetration and distribution in larger volumes of tissue. Direct injection into the scaffold pedicle fosters a natural regenerative environment, a notable advantage over bioreactors due to the comprehensive utilization of the in vivo environment. Conversely, bioreactors demonstrate superiority in mitigating cell damage and death, a benefit attributable to the precise parameter regulation.

### Application forms of AdECMs

Common application forms of dECMs to construct regenerative materials include hydrogels, large-volume scaffolds, and bioinks for 3-dimensional bioprinting, all of which have been used in muscle regeneration experiments on animals. The coating form has been successfully employed in cellular studies (Table).

**Table.** Recellularization methods, cell types, density, and application forms of AdECMs

Application forms	Materials	Cross-linking methods	Cell types	Density	Methods	Application areas	Ref
Hydrogels	Human adipose tissue	Thermal cross-linking	Human ADSCs	1.5 × 10^5^/ml	Mixed with hydrogel in vitro	Soft tissue augmentation, adipose tissue regeneration	[115]
Hydrogels	Human adipose tissue	Thermal cross-linking	Human ADSCs and human osteoinduced ADSCs	Human ADSCs 1:1 human osteoinduced ADSCs 0.5 × 10^5^/100 μl	Mixed with prehydrogel in vitro	Bone regeneration, bone defect repair	[116]
Hydrogels	Human adipose tissue	Thermal cross-linking	Human ADSCs	0.5 × 10^6^/ml (with the highest proliferation)	Mixed with prehydrogel in vitro	Soft tissue repair	[117]
Hydrogels	Human adipose tissue	Thermal cross-linking	Human ADSCs	1 × 10^6^/ml	Mixed with prehydrogel in vitro	Cell delivery, wound healing, soft tissue repair	[118]
Hydrogels	Porcine adipose tissue	Thermal cross-linking	Human ADSCs	2.5 × 10^6^/ml	Mixed with hydrogel in vitro	Soft tissue augmentation	[119]
Hydrogels	Porcine adipose tissue	Thermal cross-linking	Human ADSCs	2 × 10^7^/ml	Mixed with hydrogel in vitro	Fat grafting	[120]
Hydrogels	Human adipose tissue	Thermal cross-linking	Human ADSCs	2.4 × 10^5^/60 μl	Mixed with prehydrogel in vitro	Wound healing, tissue regeneration	[121]
Hydrogels	Human adipose tissue	Thermal cross-linking	Human ADSCs	3 × 10^6^/ml	Mixed with prehydrogel in vitro	Peripheral nerve repair and skeletal muscle regeneration	[19]
Large-volume scaffolds	Rat adipose tissue	ND	Rat ADSCs and L6	ADSCs 1:1 L6, 5 × 10^4^/50 μl	Injected into the scaffold pedicle	VML	[20]
Coating cell petri dishes	Rat adipose tissue	ND	Human ADSCs	5 × 10^4^/cm^2^	Cultured in vitro	Muscle regeneration	[18]

ND, no data

The dECM hydrogel was based on the self-assembly of collagen. The dECM was first prepared as a homogeneous solution by dissolving the dECM using enzymatic and acidic solubilization methods, followed by adjusting the solution temperature, salt ion concentration, and pH and adding cross-linking agents to induce gel formation [63–65]. Zhang et al. [18] applied a recellularized cell-derived AdECM combined with Se@SiO_2_ nanoparticles to stimulate myogenic differentiation of ADSCs. Li et al. [19] applied an AdECM to achieve repair of the sciatic nerve in Sprague Dawley rats as well as structural reconstruction and functional restoration of atrophied muscles due to sciatic nerve defects. It is important to note that the ECM treated with SDS failed to form hydrogels, whereas the ECM treated with SDC followed by Triton X-100 successfully formed 3-dimensional hydrogel scaffolds. This difference is due to the fact that SDC and Triton X-100 are milder detergents than SDS, implying a better method for balancing the removal of cells and protection of ECM components [22]. To ensure the effectiveness of decellularization in SMdECMs, biological enzymes are usually used after SDS or SDC, which may imply a more severe disruption of the original components of the ECM than that in AdECMs.

Considering that VML requires a large volume of material to repair the defect, large-volume scaffolds seem to be more appealing than hydrogels. Liang et al. developed recellularized large-volume adipose decellularized scaffolds to achieve skeletal muscle regeneration and recovery of skeletal muscle function in a rat tibialis anterior VML model. The procedure for preparing the recellularized large-volume scaffolds was reported in detail in this study. In summary, the rat inguinal adipose tissue with a pedicle, which is a branch of the femoral blood vessel, was first isolated, and then the pedicle was placed in a bioreactor, and a blunt needle was inserted into the pedicle of the bioreactor. Methods for decellularization and recellularization are summarized in previous sections [20]. When preparing SMdECM scaffolds, obtaining autologous bulk muscle scaffolds is not easy, for they are often accompanied by severe donor area morbidity and usually have an insufficient volume compared to recipient sites.

Three-dimensional bioprinting enables the construction of cell-loaded hydrogels into high-precision tissue structural scaffolds [66]. The structure and alignment of skeletal muscle bundles are important for force generation, and a harmonious structure can further enhance the recovery of function [67,68]. By combining the dECM with other natural or synthetic materials, the viscosity of the bioink is adjusted to a suitable range in order to give the bioink the right mechanical strength to form pores and maintain the structure, which is conducive to the adhesion, growth, and migration of loaded cells, suggesting a better restorative effect [69]. Three-dimensional bioprinting techniques are usually divided into jetting-based, extrusion-based, and vat photopolymerization-based techniques according to the American Society of Testing Materials [70–72]. The extrusion-based technique and vat photopolymerization can achieve a high accuracy and a large size, which is appealing when volumetric repair is needed.

## Assessment of the Structural Regeneration and Functional Recovery of Skeletal Muscle

**Fig. 3.**
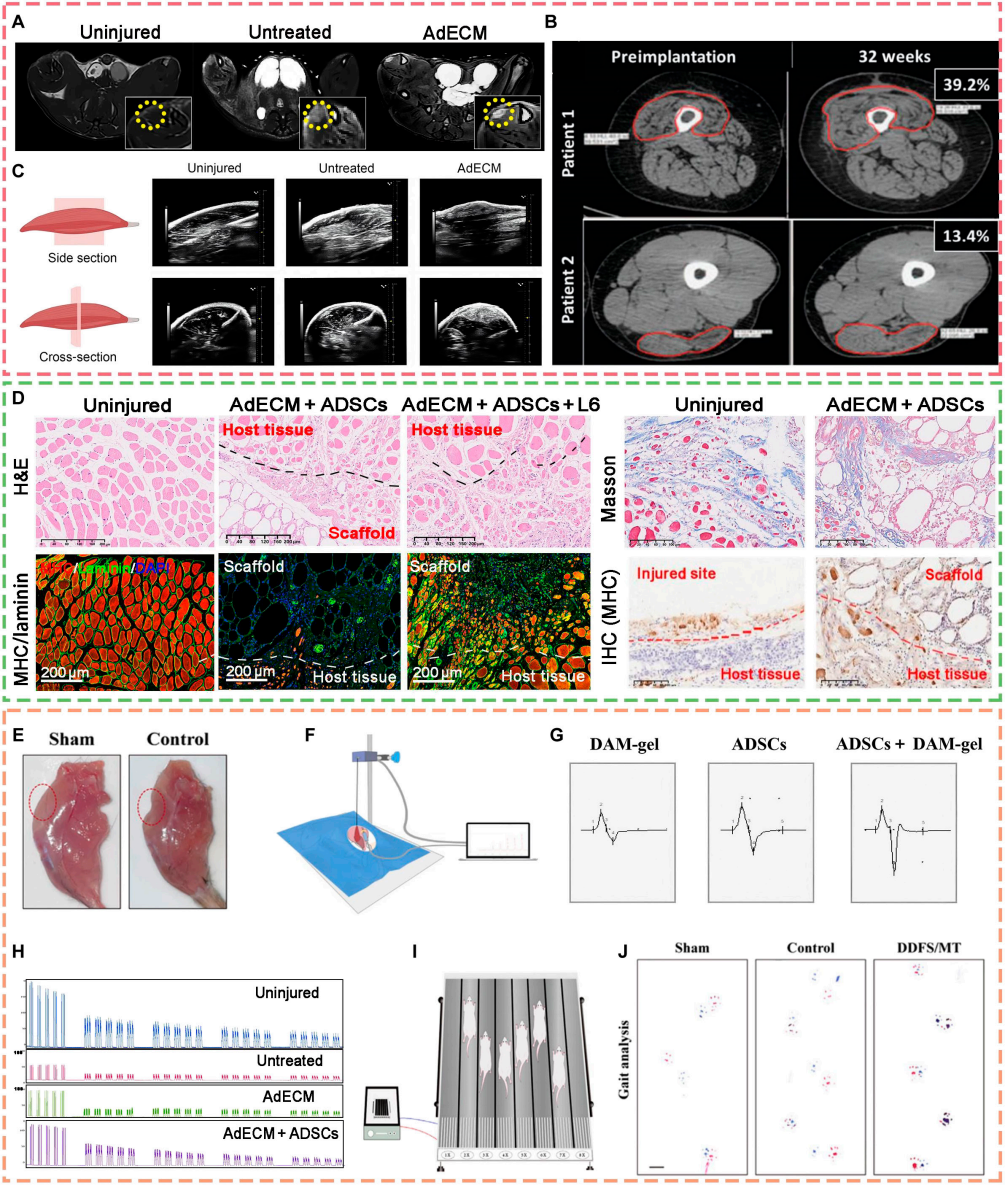
Assessment of structural regeneration and functional recovery of skeletal muscle. (A to C) Imaging assessment of muscle regeneration [20,111,112]. (D) Assessment methods of histology: hematoxylin and eosin (H&E) staining, Masson staining, immunofluorescence staining, and immunohistochemical staining of regenerated muscle [20]. (E to J) Functional assessment of regenerated muscle. (E) Appearance evaluation and weight evaluation [113]. (F and G) The scheme of the electromyography test [19,114]. (H) Fatigue and loss of the viability of muscles [20]. (I) The scheme of the treadmill experiment [114]. (J) The gait analysis experiment [113]. Reproduced with permission [19]: © 2022 Published by Elsevier B.V. Reproduced with permission [20]: © 2024 Elsevier Ltd. Reproduced with permission [112]: © 2016, The Authors. Reproduced with permission [113]: © 2022, The Authors. Reproduced with permission [114]: © 2022 Acta Materialia Inc. DAPI, 4′,6-diamidino-2-phenylindole; MHC, myosin heavy chain; DDFS/MT, decalcified fish scales loaded with myotubes.

Common structural regeneration assessments include (a) histological evaluation of myofiber regeneration, vascular regeneration, nerve regeneration, and the degree of remission of fibrosis, using hematoxylin and eosin, immunofluorescence, and immunohistochemistry staining; (b) imaging evaluation, such as magnetic resonance, ultrasonography, and computed tomography; (c) functional evaluation, such as electromyography, gait analysis, and joint mobility evaluation; and (d) weight evaluation, which is the conversion of body weight into muscle weight (Fig. 3). Table S4 summarizes the assessments of skeletal muscle regeneration according to different animal models.

## Mechanisms of AdECMs for Muscle Regeneration

### The role of AdECM components, structures, and factors in muscle regeneration processes

Basement membranes and fibronectin are important for regulating cell proliferation, differentiation, and migration and play an important role in myogenesis by promoting the movement of myoblasts to the site of injury during the regeneration phase [73,74]. The basement membrane is a specialized ECM, and its basic components include laminin, type IV collagen, and acetylheparin sulfate proteoglycan [75]. Laminin has the ability to bind cell surface receptors and acts as an initiating organizer and structural framework for basement membrane assembly [76]. Type IV collagen forms the sieve plate of the basement membrane [77]. Acetylheparin sulfate proteoglycan is a bridge connecting laminin and IV collagen [75]. According to previous studies, fibronectin, laminin, type IV collagen, and acetylheparin sulfate proteoglycan are common components of SMdECMs and AdECMs, suggesting that an AdECM has the ability to recruit myoblasts as an SMdECM. Common components of SMdECMs and AdECMs also include collagen I, which provides mechanical support to the tissue; collagen VI; and elastin, which imparts elasticity to muscle tissue, and these components are particularly important for functional muscle repair [77]. In terms of growth factors, common components of SMdECMs and AdECMs include basic fibroblast growth factor (bFGF), which promotes cell division and repair, and VEGF, which regulates angiogenesis and vascular leakage [78].

The compositional differences between AdECMs and SMdECMs are as follows: an AdECM is dominated by collagen I, and an SMdECM is dominated by collagen IV [79–81]. Collagen I promotes the proliferation and migration of myogenic cells, which play an important role in muscle regeneration, but its excessive deposition can lead to fibrosis and muscle stiffness, which may limit the regeneration efficiency of the muscle; collagen IV has a distinct advantage in promoting the migration and differentiation of myogenic cells, which can support muscle regeneration by maintaining the integrity of the basement membrane [68,82]. Collagen IV has an obvious advantage in promoting myogenic cell migration and differentiation and can support muscle regeneration by maintaining the integrity of the basement membrane. Future studies of AdECM muscle regeneration may investigate whether increasing collagen IV content can achieve more desirable muscle regeneration outcomes [68,82]. An AdECM contains more bFGF than an SMdECM, which activates muscle SCs, stimulates vascular endothelial cell proliferation and migration, improves muscle contraction, and promotes the recruitment of anti-inflammatory macrophages to modulate the inflammatory response, all of which indicate the superiority of AdECMs in muscle formation [80,83,84]. In addition, AdECMs contain abundant immunomodulatory proteins, such as interleukin 10, whereas SMdECMs contain fewer immunomodulatory factors [79,81]. Furthermore, AdECMs contain higher levels of matrix metalloproteinases (MMPs), such as MMP2 and MMP9 [12,80]. The abundance of anti-inflammatory factors and ECM remodeling factors may reduce fibrosis and inflammatory responses.

### Skeletal muscle regeneration mechanisms of recellularized AdECMs

#### ADSCs promote SC myogenic differentiation in recellularized AdECMs

SCs located in the niche between the basal lamina and the sarcolemma are able to mobilize rapidly after muscle injury, are recruited to the site of injury, and are activated by the surrounding environment and other SCs, and their self-renewal proliferation not only maintains the stem cell population but also provides a large number of myogenic cells, which proliferate, differentiate, fuse, and lead to new muscle fiber formation and reconstitution of the functional contractile apparatus [25]. SCs express a variety of proteins, including the transcription factors myogenic differentiation (MyoD), Pax7, myogenic factor 5, and Pax3 [65,85].

**Fig. 4.**
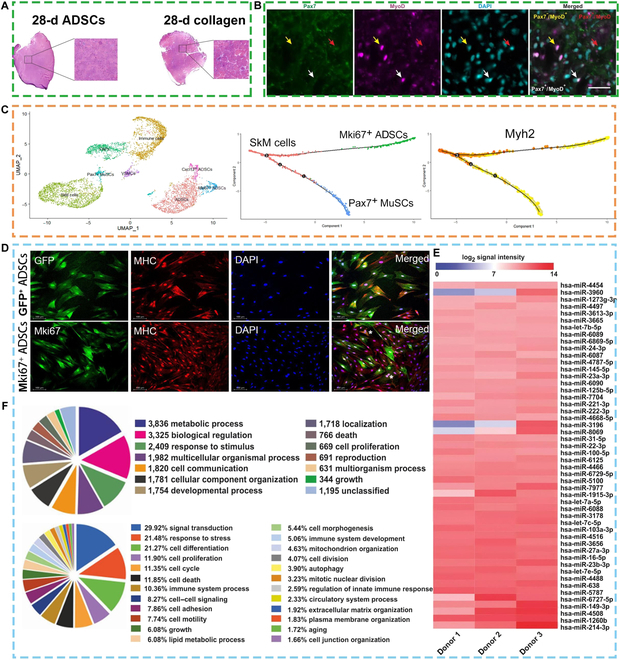
Skeletal muscle regeneration mechanisms of recellularized AdECM. (A) Representative cross-sections of tibialis anterior (TA) muscles following crush injury with ADSC or collagen transplantation [86]. (B) Immunohistochemical staining and quantification of satellite cell myogenic progression; scale bar: 20 μm [87]. (C and D) Myoblasts promote myogenic differentiation of ADSCs: cluster composition of the AdECM + ADSCs + L6 group. The monocle pseudotime analysis of Mki67^+^ ADSCs [20]. (E) Heatmap of the top 50 microRNA (miRNA) species hits within the ADSC secretome as determined by miRNA array analysis [87]. (F) Database mining and functional analysis mapping the biological processes of the 22,903 messenger RNA (mRNA) target hits from the miRNA array [87]. Reproduced with permission [86]: © 2018, The Authors. Reproduced with permission [87]: © 2019, The Authors. Reproduced with permission [20]: © 2024 Elsevier Ltd. GFP, green fluorescent protein; MyoD, myogenic differentiation; UMAP, uniform manifold approximation and projection; VSMCs, vascular smooth muscle cells; Cxcl12, chemokine (C-X-C motif) ligand 12; Myh2, myosin heavy chain 2.

The autoregenerative capacity of SCs is limited and cannot meet the needs of large muscle defects, often resulting in scarring. Previous studies have used ADSCs as recellularization materials in muscle defect models and achieved considerable muscle repair. ADSCs may affect SC differentiation by directly acting on SCs or modulating the ECM, but are not directly involved in the composition of muscle fibers. Gorecka et al. [86] conducted relevant experiments to explore the mechanism of action of ADSCs affecting muscle regeneration. In vitro ADSCs and SCs co-culture experiments showed that ADSCs could promote SCs to enter an activated state and express Pax7^+^ MyoD^−^ [86]. Optical projection imaging and histological pictures suggested that ADSCs were not directly involved in composing muscle fibers, but could increase the diameter of muscle fibers (Fig. 4A and B) [86]. This study also showed high expression of transforming growth factor beta 1 in the ADSC group, suggesting that it may be involved in the ECM remodeling process, which is important in myogenic differentiation. Subsequent animal experiments confirmed that ADSCs significantly increased skeletal muscle contractility [86]. Researchers have further investigated the specific role of ADSCs in the process of muscle formation and concluded that ADSCs promote muscle regeneration mainly through the secretion of extracellular vesicles and soluble substances. One study analyzed the ADSC secretome, which contains the proangiogenic paracrine factors hepatocyte growth factor, bFGF, IGF-1, and VEGF miRNAs (miR-23a and miR-23b), and the miRNAs that play a role in regulating immunity and anti-inflammation (Fig. 4E and F) [87]. Based on the research experience on ADSC exosomes promoting regeneration in other tissues, the investigators hypothesized that ADSC exosomes promote muscle regeneration through activation of the wingless/integrated (Wnt) pathway, the mitogen-activated protein kinase (MAPK) pathway, and the phosphatidylinositol 3-phosphate kinase pathway. The kinase/protein kinase B (PI3K/Akt) pathway and the tetanus kinase/signal transducer and activator of transcription (JAK/STAT) pathway promote cell proliferation, myogenic differentiation, and muscle regeneration [85].

#### Myoblasts promote myogenic differentiation of ADSCs in recellularized AdECMs

It has also been shown that in promoting myogenesis, ADSCs and their secretome can not only promote myogenic differentiation of muscle SCs but also have the potential for myogenic differentiation themselves. However, despite the multiple differentiation potential and easy accessibility of ADSCs, the low efficiency of myogenic differentiation of ADSCs, with only 15% of ADSCs differentiating into myoblasts in in vitro cultures, has limited their application in the muscle tissue engineering field [88]. Considering that the myogenic effect of muscle SCs remaining in the injured tissue alone is not sufficient for the repair of VML, combined with the fact that muscle SCs are difficult to obtain, it is important to look for means that can promote myogenic differentiation of ADSCs in VML.

Studies have shown that co-culture of ADSCs with myoblast increases the efficiency of myogenic differentiation of ADSCs. Liang et al. found that L6 cells induced the transformation of ADSCs into a new subpopulation of cells with high expression of Mki67, CD34, and CDK1 genes, which are known to have a greater capacity for directed myogenic differentiation in AdECMs (Fig. 4C and D). This study further elucidated the specific role of the Mki67^+^ ADSC population and revealed that matrix interacting molecules 1 and 2 (Stim1 and Stim2), the major store of Ca^2+^ entry mediator proteins in skeletal muscle cells, are present in Mki67^+^ ADSCs, skeletal muscle (SkM) cells, and Pax7^+^ muscle SCs. In addition, in SkM cells and Mki67^+^ ADSCs, splicing factor splicing factor 1 (srsf1) was also detected in SkM cells and Mki67^+^ ADSCs [20].

### AdECMs inhibit inflammatory responses and promote angiogenesis and neuralization by modulating the local microenvironment

**Fig. 5.**
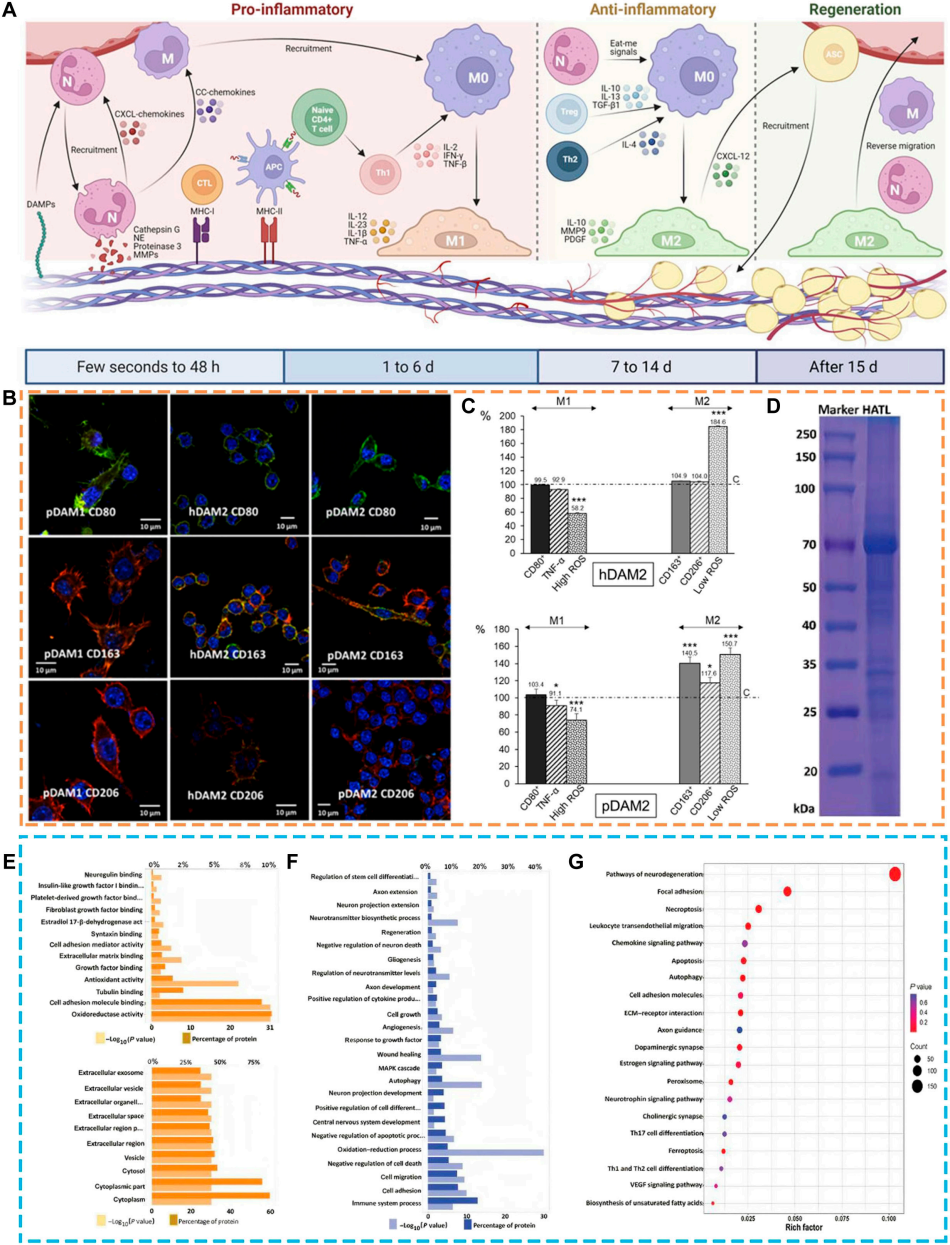
AdECMs inhibit inflammatory responses and promote angiogenesis and neuralization by modulating the local microenvironment. (A) Schematic diagram of the host inflammatory cell implantation into the acellular adipose matrix [101]. (B) M1- and M2-specific markers observed by confocal microscopy in RAW-264.7 macrophages after 24 h of culture on control surface (CTRL) and human or porcine decellularized adipose matrices (DAMs) obtained by organic solvent treatment [93]. (C) Percentage of pro-inflammatory M1 RAW-264.7 macrophages (characterized by CD80^+^, tumor necrosis factor alpha [TNF-α], and high reactive oxygen species [ROS]) and reparative M2 macrophages (characterized by CD163^+^, CD206^+^, and low ROS) after 24 h of culture on human (h) and porcine (p) DAMs obtained by treatment with organic solvents; *P* < 0.05 was considered statistically significant (*P < 0.05; ***P < 0.001) [93]. (D to G) Proteomic analysis of adipose tissue lysate showing that the AdECM is neuroprotective and promotes nerve regeneration and functional recovery. (D) The protein bands distribution of adipose tissue lysate. (E) Gene Ontology (GO) analysis of the proteins’ functional enrichment based on cellular components, molecular function (F), and biological process (G) [104]. Reproduced with permission [101]: © 2022 Liu, He, and Lu. Reproduced with permission [93]: © 2021 by The Authors. Reproduced with permission [104]: © 2023 Wiley-VCH GmbH. DAMPs, damage-associated molecular patterns; MMPs, matrix metalloproteinases; IL, interleukin; IFN-γ, interferon gamma; TGF, transforming growth factor; Th1, T helper cell 1; Th2, T helper cell 2; PDGF, platelet-derived growth factor; CC, CC-chemokines; NE, neutrophil elastase; APC, antigen-presenting cells; HATL, human adipose tissue lysate-based hydrogel.

AdECMs inhibit the inflammatory microenvironment. Inflammatory responses in decellularized materials arise mainly from incomplete decellularization, with DNA fragments being the most common cellular component causing inflammatory responses [50,89,90]. As mentioned earlier, the natural properties of skeletal muscle lead to its low decellularization efficiency when ensuring enough residual ECM composition for cell adhesion and proliferation, which may have contributed to the high inflammatory response and fibrosis. It has been shown that AdECMs of human and porcine origins play an important role in balancing the pro-inflammatory M1 and anti-inflammatory M2 phenotypes of macrophages (Fig. 5B and C) [91–93]. AdECMs significantly down-regulated the expression of the surface molecule CD80 of M1 and reduced tumor necrosis factor alpha release while inhibiting oxidative stress, as well as up-regulating the expression of the surface molecules CD163 and CD206 of M2 [93]. Regulatory T (Treg) cells are immunomodulatory cells that play a key role in maintaining immune homeostasis [94]. In general, T helper cell 1 (Th1) polarization promotes pro-inflammatory responses, whereas T helper cell 2 (Th2)/Treg polarization is involved in graft acceptance and constructive remodeling responses; reduced Th1:Th2 levels in grafts may facilitate in vivo retention and regeneration of dECMs [95,96]. It has been shown that increased levels of Treg cells in methoxypolyethylene glycol-modified AdECMs induce lipogenesis and M2 polarization [97]. In addition, damage-associated molecular patterns on AdECM as well as regenerating blood vessels can negatively regulate the inflammatory response by recruiting neutrophils to further promote M2 polarization [97].

The AdECM microenvironment facilitates angiogenesis. AdECMs promote M2 polarization, and the M2d subtype has been shown to increase VEGF release [98]. Neutrophils secrete serine proteases, elastase, and histone G, as well as MMPs, which can cleave collagen, fibronectin, elastin, proteoglycans, and laminin in the ECM, which in turn breaks down connective tissues and induces angiogenesis and matrix remodeling (Fig. 5A) [99–101].

AdECMs are neuroprotective and promote nerve regeneration and functional recovery. Experiments on the treatment of spinal cord injury with AdECMs have confirmed that AdECMs can reduce chronic neurodegeneration by inhibiting neuroinflammation and neuronal apoptosis [102–104]. In addition, AdECMs were shown to have the effect of recruiting neural progenitor cells, promoting the differentiation of neural progenitor cells to neurons and oligodendrocytes and reducing glial scar formation. Up-regulation of doublecortin X and β-tubulin expression indicates neuronal maturation, NF-200 staining indicates axonal regeneration, and glial fibrillary acidic protein staining indicates a reduction in glial scarring, which is achieved by a reduction in glial cell activation induced by various inflammatory cytokine-mediated STAT3 pathways [104–106]. In addition, the expressions of Trpc5, ChAT, Scn1a, and Hoxd10 were significantly higher than those of the control group by detecting genes related to motor neuron development, suggesting that AdECMs facilitate the restoration of motor function in innervated limbs (Fig. 5H and G) [104].

## Prospects and Conclusions

At present, both SMdECMs and AdECMs have limitations in muscle regeneration. They face problems such as muscle regeneration effects that still need to be improved, the inability to completely decellularize, the loss of ECM properties, and the difficulty of reproducibility due to the differences in dECMs from different batches. Regarding the myogenic effect, both exhibit fibrosis and poor myofiber alignment. Additionally, the absence of a comprehensive functional evaluation in several trials hindered the assessment of muscle regeneration recovery. Concerning residual cellular components, AdECMs may not achieve complete removal, potentially resulting in residual cellular debris and triggering an immune response or affecting the regenerative effect. It is technically more challenging to achieve complete decellularization of SMdECMs than that of AdECMs due to their more intricate structure and the complexity of completely removing cellular components through vascular perfusion. Regarding the retention of ECM properties, certain differences exist between AdECMs and the natural environment of skeletal muscle regeneration. While SMdECMs demonstrate a higher retention of natural ECM components, they are also susceptible to damage, albeit to a certain extent, due to the more intensive decellularization method employed.

Currently, there are few studies on the myogenesis of AdECMs, and although the potential of AdECMs for myogenesis has been demonstrated, more experimental validation is needed before a unified conclusion can be reached.

AdECM decellularization methods are relatively abundant and mature, ensuring decellularization efficiency with as little disruption of the ECM components as possible, and ensuring that the structure of the ECM is conducive to the growth, adherence, and migration of myoblasts. Compared with SMdECMs, AdECMs have a relatively single cell type and method of recellularization, and subsequent studies can improve the myogenic effect of AdECMs by enriching the cell types and methods of recellularization.

In terms of application forms, large-volume scaffolds are more ideal for VML repair, and decellularized adipose tissue with vascular tips is easy to perfuse, so the decellularization efficiency is high and the recellularization is uniformly distributed. Hydrogels are still irreplaceable for small-volume muscle defects such as those in the maxillofacial region. The protective effect of the natural microstructure of hydrogels on cells can be further improved by adding natural or synthetic polymers to the hydrogels, and the addition of anti-inflammatory biofactors can inhibit the fibrosis of the muscle-forming process. Three-dimensional bioprinting inks are a promising material for applications where the loaded cells can be distributed homogeneously and maintain a high degree of precision of the tissue structure and can address the inability of large-volume scaffolds to achieve regenerative muscle fiber alignment. Future research could focus on AdECM muscle-forming bioinks.

The assessment of the effect of muscle formation includes morphological and functional assessment, which varies according to different animals and different modeling sites. Multidimensional evaluation of muscle formation necessitates concurrent morphological and functional assessments, customized for distinct model systems. Current modeling methods are mostly limited to limbs, abdomen and diaphragm, and there are fewer studies on the modeling of muscles in the head and face, which can be focused on in the future studies.

The mechanism of myogenesis in AdECMs and SMdECMs is not sufficiently researched, and the current consensus is that the exosome component of ADSCs promotes myogenic differentiation of SCs and regulates the ECM to create a suitable microenvironment for myogenesis, while some studies have pointed out that it is possible to utilize the potential of myogenic differentiation of ADSCs themselves. In the future, targeted studies on the mechanism of myogenic differentiation are needed to provide effective guidance for the subsequent experiments.

## Data Availability

All data included in this study are available upon request by contact with the corresponding authors.

## Supplementary Materials

Supplementary 1 Figs. S1 and S2 Tables S1 to S4 References [100–114,122–134]
