# Supplementary material for: Adipose Decellularized Matrix: A Promising Skeletal Muscle Tissue Engineering Material for Volume Muscle Loss
Source: Biomater Res. 2025 Apr 17;29:0174. doi: 10.34133/bmr.0174 (PMC12003953; doi:10.34133/bmr.0174)
Supplement: Supplementary 1 — Figs. S1 and S2 Tables S1 to S4 References [100–114,122–134] [file bmr.0174.f1.docx]

**Supporting Information**

**Adipose Decellularized Matrix: A Promising Skeletal Muscle Tissue Engineering Material for Volume Muscle Loss**

**Zimo Wang^1#^, Wei Liang^1#*^, Rigele Ao^1^, Yang An^1*^**

^1^Department of Plastic Surgery, Peking University Third Hospital, 49 North Garden Road, Haidian District, Beijing, 100191, China.

^#^ The authors contributed equally to this work.

**^*^Corresponding Author**

[liangwei8790@163.com](mailto:liangwei8790@163.com) (Wei Liang)

anyangdoctor@163.com (Yang An).

**Figure S1. Regeneration Process of Skeletal Muscle.** The diagram illustrates the 5 stages of regeneration after muscle damage and the myogenic and circulatory-derived cells involved.


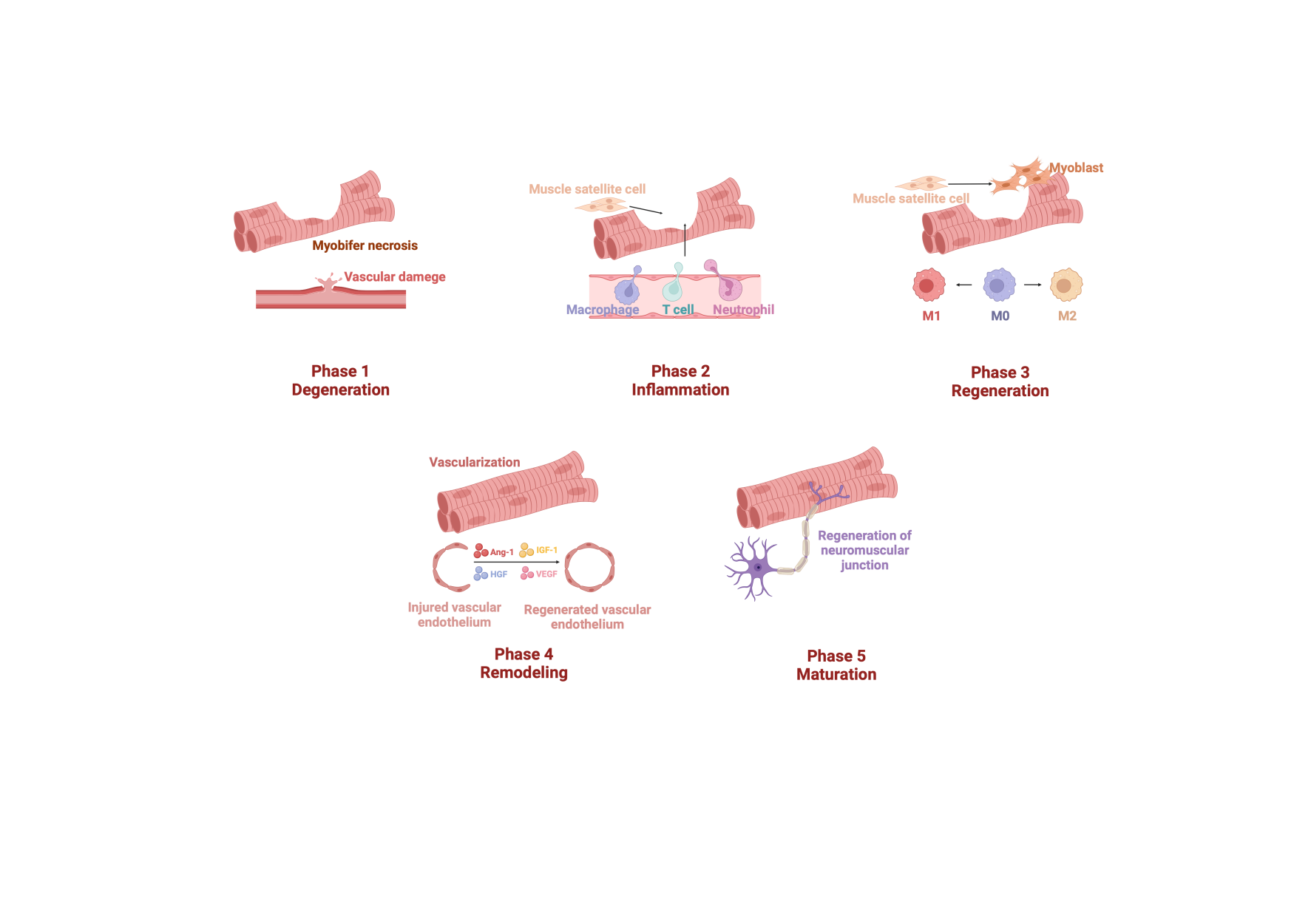


**Figure S2. Decellularizition methods to prepare AdECM and recellularization methods of AdECM and SMdECM. A.** Popular methods and reagents used for physical, chemical and biological enzymatic decellularization to prepare AdECM. **B.** Recellularization methods of AdECM reported in previous studies, including culturing in vivo in the hydrogel, mixing in vitro with pre-hydrogel before before injection, and being injected into scaffold pedicle with myoblasts. **C.** Other recellularization methods reported in study of SMdECM.


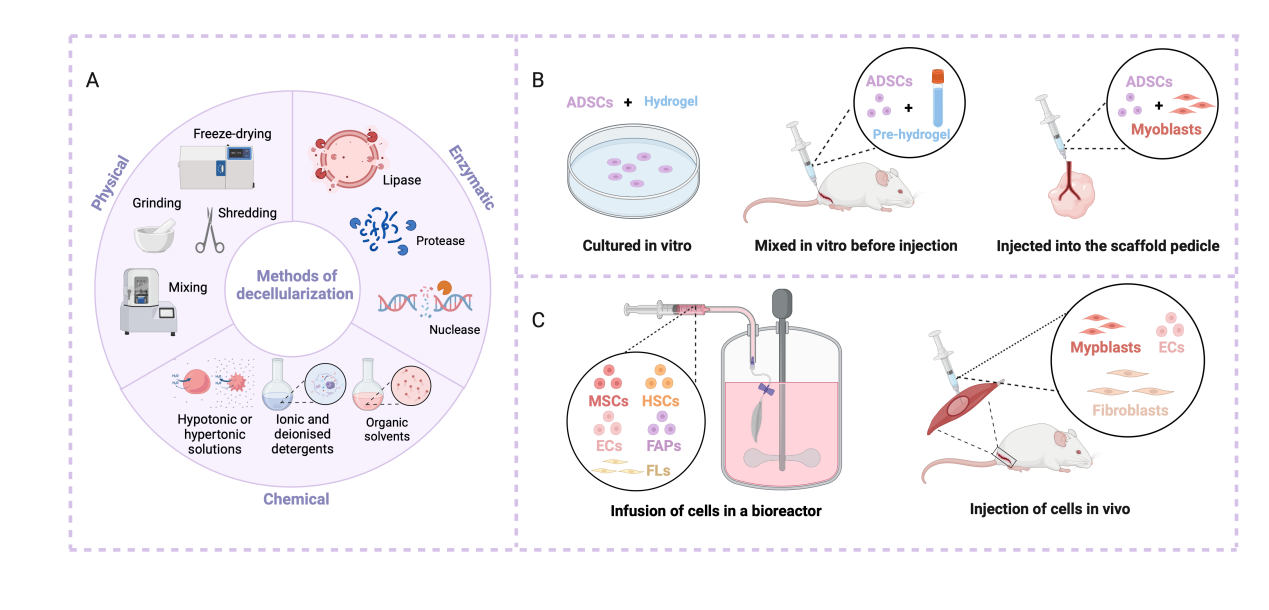


**Table S1. Components comparison of SMdECM and AdECM**

| **Components** | **Function** | **SMdECM（√/×）** | **AdECM（√/×）** |
| --- | --- | --- | --- |
| **Collagen** | | | |
| collagen I | Coarse diameter and rich content，determining the firmness and strength of the collagen network. | √ | √ |
| collagen II | High strength，providing organizational integrity and stress resistance |  |  |
| collagen III | Highly flexible, improving tissue softness and regulating mechanical properties of type I collagen | √ | √ |
| collagen IV | The main components of the basement membrane form the basement membrane sieve plate,providing the supporting force | √ | √ |
| collagen V | Structural support for collagen I and III | √ |  |
| collagen VI | Mechanical support;  Maintenance of SC niche | √ | √ |
| **glycosaminoglycan** | | | |
| Acetylheparin sulphate | Binding of growth factors;  Maintenance of SC niche | √ | √ |
| **proteoglycan** | | | |
| SLRPs | Regulator of collagen I  fibrillogenesis | √ | √ |
| **Elastin** | Elastic fibre component, giving tissue elasticity, basement membrane component | √ | √ |
| **Fibronectin** | Cell adhesion to the ECM, affecting cell  survival, spreading, migration, proliferation, differentiation, etc. | √ | √ |
| **Laminin** | Binding to the cell membrane integrin receptor, critical for basement membrane assembly | √ | √ |
| **Growth factor** |  |  |  |
| bFGF | Promoting cell division and repair | √ | √ |
| VEGF | Regulating angiogenesis and vascular leakage | √ | √ |
| PDGF | Promoting cell division and repair | √ | √ |
| TGF-β | Regulating cell growth, differentiation and immune function | √ | √ |
| IGF-1 | Mediating growth hormone stimulation, regulating tissue growth and development, and playing an important role in muscle size and strength, maintenance of body composition and regulation of nutrient metabolism | √ | √ |
| angiopoietin-1 | complementing and coordinating with VEGF during angiogenesis and late vascular maturation | √ | √ |

Abbreviation: Skeletal muscle decellularized matrix (SMdECM); Adipose decellularized matrix (adECM); Small Leucine-Rich Proteoglycans (SLRPs); Basic fibroblast growth factor (bFGF); Vascular endothelial growth factor (VEGF); Platelet-derived growth factor (PDGF); Transforming growth factor-β (TGF-β); Insulin-like growth factor-1 (IGF-1).

**Table S2. Sources of AdECM**

| **Sources** | **spieces** | **ADSCs/ adipose origin** | **Ref** |
| --- | --- | --- | --- |
| **dECM deposited by ADSCs** | SD rats | adipose tissue throughout the body | ^[18]^ |
| **adipose-tissue derived dECM** | Human donor | breast | ^[111, 112, 117, 118]^ |
|  |  | abdomen | ^[107, 109, 110, 113, 118-124]^ |
|  |  | thigh | ^[107, 109, 110, 113, 120, 123, 124]^ |
|  | SD rats | groin region | ^[19, 20]^ |
|  | C57BL/6 mice | Subcutaneous inguinal | ^[63]^ |
|  | Porcine | adipose tissue throughout the body | ^[125]^ |

**Table S3. Physical, chemical and enzymatic decellularization methods of AdECM**

| Decellularization methods | | | Ref |
| --- | --- | --- | --- |
| Physical | Chemical | Enzymatic |  |
| 4 freeze-thaw cycles(-80℃);  Agitation;  grinding | 0.5% SDS at 46℃ for 20 min; lyophilized and immersed in xylene (1:10 w/v) for 17 min | 0.05% trypsin/0.05 mM EDTA(1:1v/v)at 37 ℃ for 90 min ; DNAse (30 lg/ml DNAse in  1.3 mM MgSO4 and 2 mM CaCl2) overnight at 37℃ | ^[120, 121, 126]^ |
| 3–5 freeze-thaw cycles from 37℃ to -80℃ | 1% Triton-X100 at 37℃ for 1h; 100% isopropanol overnight at 37 ℃ | Equal volume of 1.0 mol/L NaCL, 100U/mL DNase and 100μg/mL RNase overnight at 37℃ | ^[122]^ |
| Agitation | 1% SDS for  2 days; 2.5 mM sodium deoxycholate for 2 days | 500 U lipase and colipase for 2 days | ^[107]^ |
| 3 freeze-thaw cycle;  grinding | Isopropanol for 48 h;  1% triton X-100 for 48 h | 0.25% trypsin for 2h | ^[87, 109, 127-129]^ |
| Homogenizing;  grinding | 0.5% SDS for 4h; 100% isopropanol for 2h |  | ^[111]^ |
| homogenizing | 0.5% SDS for 4h；100% isopropanol for 2h | 1mg/mL pepsin | ^[112]^ |
| Homogenizing;  grinding | 1% SDS in DI water or 2.5mM sodium deoxycholate in 1xPBS for 48 h | 500U colipase | ^[117]^ |

Abbreviation: Sodium dodecyl sulfate (SDS); Ethylene Diamine Tetraacetic Acid (EDTA); Deoxyribonuclease (DNAse); ribonuclease (RNase); (DI). Deionized water.

**Table S4. Assessment of structural regeneration and functional recovery of skeletal muscle**

| Animal | Anatomical site | Construction Form | Recellularization | Histology | Imaging | Weight | Function | Ref. |
| --- | --- | --- | --- | --- | --- | --- | --- | --- |
| Mouse | latissimus dorsi muscle | hydrogel | N/A | H&E and modified VerHoff Gieson stain for collagen and elastin and muscle fibers, H&E and immunohistochemi  cal staining for inflammation | N/A | N/A | N/A | ^[51]^ |
|  | Diaphragm | Hydrogel; large-volume scaffolds | N/A | H&E staining, Masson’s trichrome, DAPI, immunofluorescent staining for inflammatory response, myogenic cell activation, angiogenesis and nerve attraction and re-growth | CT scan and lung area | N/A | electromyogram | ^[45-47]^ |
|  | Tibialis anterior | large-volume scaffolds | Human SCs and MRCs | H&E staining for muscle fibers, FACS plots and immunofluorescence for myogenesis, angiogenesis and fibrosis | N/A | Yes | electromyogram | ^[130]^ |
| Rat | latissimus dorsi muscle | Large-volume scaffolds | N/A | H&E staining, immunofluorescent staining for myogenesis and angiogenesis | N/A | Yes | electromyogram | ^[12]^ |
|  | quadriceps muscle | Large-volume scaffolds | HASCs, 1*10^5/cm^2^ | H&E staining, immunohistochemical staining for myogenesis | N/A | N/A | N/A | ^[131]^ |
|  | Tibialis anterior | Large-volume scaffolds | ADSCs (2.5*10^6/50μL）and L6(2.5*10^6/50μL)；BMSCs (1*10^6/30μL) | H&E, immunohistochemistry , Masson, immunofluorescence and snRNA-seq for myogenesis | MRI and ultrasound of muscles | Yes | Electromyogram, Gait analysis, ROM of ankle joint | ^[20, 131]^ |
|  | gastrocnemius | Large-volume scaffolds | BMSCs(1.5-2.0*10^6/300μL) | Masson’s trichrome and immunofluorescence for myogenesis and angiogenesis | N/A | N/A | electromyogram | ^[132, 133]^ |
|  | extensor digitorum longus | Large-volume scaffold | SCs and fibroblasts | bungarotoxin and synaptophysin staining for percentage of neuromuscular junctions, H&E staining Masson’s Trichrome staining, immunofluorescence for myogenesis and angiogenesis | N/A | Yes | electromyogram | ^[31]^ |
| Rabbit | rectus abdominis muscle | Large-volume scaffold | N/A | H&E staining for myogenesis and angiogenesis | N/A | N/A | N/A | ^[54]^ |
|  | external oblique muscle | Large-volume scaffold | N/A | hematoxylin and picro-Ponseau for myogenesis, fibrous tissue and angiogenesis | N/A | N/A | N/A | ^[16]^ |
| Dog | diaphragm | Large-volume scaffold | N/A | H&E staining for myogenesis, angiogenesis and inflammation | CT | N/A | electromyogram | ^[134]^ |

Abbreviation: satellite cells (SCs); muscle resident cells (MRCs); human adipose-derived stem cells (HASCs); [bone marrow mesenchymal stromal cells](https://pubmed.ncbi.nlm.nih.gov/35047499/) (BMSCs); hematoxylin and eosin (H&E); computed tomography (CT); magnetic resonance imaging (MRI); range of motion (ROM).
